# Site-Specific Glycosylation of Recombinant Viral Glycoproteins Produced in *Nicotiana benthamiana*

**DOI:** 10.3389/fpls.2021.709344

**Published:** 2021-07-22

**Authors:** Emmanuel Margolin, Joel D. Allen, Matthew Verbeek, Michiel van Diepen, Phindile Ximba, Rosamund Chapman, Ann Meyers, Anna-Lise Williamson, Max Crispin, Edward Rybicki

**Affiliations:** ^1^Division of Medical Virology, Department of Pathology, Faculty of Health Sciences, University of Cape Town, Cape Town, South Africa; ^2^Wellcome Trust Centre for Infectious Disease Research in Africa, University of Cape Town, Cape Town, South Africa; ^3^Institute of Infectious Disease and Molecular Medicine, Faculty of Health Sciences, University of Cape Town, Cape Town, South Africa; ^4^Biopharming Research Unit, Department of Molecular and Cell Biology, University of Cape Town, Cape Town, South Africa; ^5^School of Biological Sciences, University of Southampton, Southampton, United Kingdom

**Keywords:** glycoprotein, glycosylation, occupancy, folding, processing, molecular pharming

## Abstract

There is an urgent need to establish large scale biopharmaceutical manufacturing capacity in Africa where the infrastructure for biologics production is severely limited. Molecular farming, whereby pharmaceuticals are produced in plants, offers a cheaper alternative to mainstream expression platforms, and is amenable to rapid large-scale production. However, there are several differences along the plant protein secretory pathway compared to mammalian systems, which constrain the production of complex pharmaceuticals. Viral envelope glycoproteins are important targets for immunization, yet in some cases they accumulate poorly in plants and may not be properly processed. Whilst the co-expression of human chaperones and furin proteases has shown promise, it is presently unclear how plant-specific differences in glycosylation impact the production of these proteins. In many cases it may be necessary to reproduce features of their native glycosylation to produce immunologically relevant vaccines, given that glycosylation is central to the folding and immunogenicity of these antigens. Building on previous work, we transiently expressed model glycoproteins from HIV and Marburg virus in *Nicotiana benthamiana* and mammalian cells. The proteins were purified and their site-specific glycosylation was determined by mass-spectrometry. Both glycoproteins yielded increased amounts of protein aggregates when produced in plants compared to the equivalent mammalian cell-derived proteins. The glycosylation profiles of the plant-produced glycoproteins were distinct from the mammalian cell produced proteins: they displayed lower levels of glycan occupancy, reduced complex glycans and large amounts of paucimannosidic structures. The elucidation of the site-specific glycosylation of viral glycoproteins produced in *N. benthamiana* is an important step toward producing heterologous viral glycoproteins in plants with authentic human-like glycosylation.

## Introduction

Plant-based expression systems are gaining increased attention for the production of recombinant proteins, including subunit vaccines and therapeutic monoclonal antibodies (Sack et al., 2015; Group et al., 2016). This is largely driven by the potential for more rapid implementation timelines and capacity for rapid production scale-up when compared to established expression systems. The upfront capital investment for a plant-based manufacturing facility is also considerably lower than for mammalian cell production, and the production costs for the raw material are similarly reduced (Nandi et al., 2016). These features are appealing to low-and middle-income countries where biomanufacturing capacity is urgently needed, but is mostly absent (Margolin et al., 2020a). The inherent ease of biomass production also offers the prospect of rapid vaccine production in response to pandemic viral outbreaks (Margolin et al., 2018), as has recently been demonstrated by Medicago Inc., in Canada for a SARS-CoV-2 virus-like particle (VLP) vaccine candidate (Ward et al., 2020a). However, products from plant-based expression systems may not always display desired critical quality attributes: for example, when a biologic requires extensive post-translational modifications, that are not performed in plants as in other cell types, there is consequently a need to further develop plant-based expression technologies to better support the production of complex pharmaceutical products such as glycosylated biologics (Margolin et al., 2020b).

Ideally, a plant-based manufacturing platform should support high yields of recombinant proteins that are appropriately post-translationally processed and biologically active. Encouraging findings have been described for many targets, including the clinical development of influenza and SARS-CoV-2 VLP-based vaccines, and the emergency use of plant-made antibody cocktails for treatment of Ebola virus infection (Landry et al., 2010; Ward et al., 2014; Group et al., 2016; Pillet et al., 2016). However, in many other cases expression yields remain a challenge, and the plant cellular machinery may also not support extensive post-translational modifications for complex biopharmaceutical targets (Margolin et al., 2020c). Virion envelope glycoproteins are amongst the most challenging targets to produce in plants, and the inability to consistently produce native-like glycoproteins from diverse targets constrains the use of the platform for vaccine development against more complex pathogens.

The requirement for “native-like” glycosylation for appropriate immunogenicity is dictated by the antigen of interest rather than being an absolute, and it is worth noting that many targets do not require native glycosylation to elicit appropriate immunogenicity. It is also noteworthy that oligomannose-type glycans are often enriched in viral glycoproteins, such as reported for HIV-1 (Doores et al., 2010; Bonomelli et al., 2011) and SARS-CoV-2 (Watanabe et al., 2020), but are generally less abundant in plants and mammalian systems. Plant-produced influenza hemagglutinin (HA)-based VLP vaccines, for example, have been reported to be efficacious and well tolerated in phase 3 clinical trials despite the presence of typical plant-derived glycans (Ward et al., 2020b). Similarly, an insect-cell produced SARS-CoV-2 spike nanoparticle vaccine has recently been reported to be protective against COVID-19 (Keech et al., 2020), and the presence of species-specific glycosylation does not appear to negatively impact the folding or immunogenicity of this antigen. In contrast, native-like glycosylation is widely regarded as an essential requirement for an HIV envelope-based vaccine, and subtle differences in glycosylation can dictate the quality of the immune response (Behrens and Crispin, 2017). Additionally, glycosylation is intrinsically linked to protein folding, and aberrant glycosylation and particularly under-glycosylation could compromise the efficient production of viral glycoproteins in a heterologous system (Margolin et al., 2020b).

Remodeling the secretory pathway has been proposed as a strategy to improve the production of complex biologics in plants, by providing a tailor-made environment to support their maturation (Margolin et al., 2020b,e). In the case of viral glycoproteins this would involve eliminating undesired plant-specific glycosylation and expressing the required heterologous cellular machinery to support appropriate glycosylation, folding and processing of the proteins as dictated by the antigen of interest (Meyers et al., 2018; Margolin et al., 2020c,e). Accordingly, the co-expression of human chaperone proteins was reported to improve the yields of several viral glycoproteins in plants and supported the production of antigens that could not previously be produced in the system (Meyers et al., 2018; Margolin et al., 2020c,d). Proteolytic processing of a viral glycoprotein has also been achieved–this time by the co-expression of human furin (Margolin et al., 2020c). However, similar strategies to produce viral glycoproteins with “human-like” glycosylation in plants have not been reported, although considerable progress has been made in this regard for antibodies and other biologics (Montero-Morales and Steinkellner, 2018).

The expression of glycoproteins in plants leads to the formation of three major glycoforms–namely complex, paucimannosidic and Lewis A structures–which are a feature of the expression system and distinguish plant-produced proteins from those made in conventional expression platforms (Montero-Morales and Steinkellner, 2018). Complex-type glycans containing α1,3-fucose and β1,2-xylose are the most abundant glycan species, and are generated in the Golgi apparatus by α1,3-fucosyltransferase and β1,2-xylosyltransferase, respectively (Wilson et al., 2001). Although these extensions do not occur in mammalian cells and might therefore be expected to appear foreign to mammalian immune systems, they do not seem to impair the immunogenicity of plant-produced influenza vaccines in humans, nor is there any evidence that they pose safety concerns in individuals with pre-existing plant allergies (Ward et al., 2014). However, the observation that these vaccines induced transient IgG and IgE responses to glyco-epitopes cannot be discounted (Ward et al., 2014) and some concerns remain, especially in the context of more heavily glycosylated proteins or where repeated administration may be necessary (Margolin et al., 2020b). Paucimannosidic glycans, comprising truncated structures where the terminal N-acetylglucosamines have been removed from the glycan core, also occur in plants (Liebminger et al., 2011). These are observed in vacuolar and extracellular glycoproteins due to processing by β-hexosaminidases that are localized to these sites (Shin et al., 2017). These modifications are generally undesirable for human pharmaceuticals as they may promote rapid protein clearance following immunization if they are recognized as foreign (Yang et al., 2015). The final plant glycan structure is the Lewis A epitope which is defined by the presence of β1,3-galactose and α1,4-fucose extensions of the terminal N-acetylglucosamines of the glycan (Fitchette-Laine et al., 1997; Wilson et al., 2001).

Recent studies have also suggested that certain proteins may have lower levels of glycan occupancy when produced in plants, but is presently unclear how widespread this phenomenon is (Jarczowski et al., 2016; Castilho et al., 2018; Göritzer et al., 2020; Singh et al., 2020). Encouragingly, a recent study reported similar glycan occupancy for recombinant human cytomegalovirus glycoprotein B when produced in *Nicotiana tabacum* BY-2 and CHO cells. However, under-glycosylation could account for the poor yields of other glycoproteins in the system, given that inadequate glycan occupancy could impair protein folding and result in the degradation of aberrantly folded protein (Wormald and Dwek, 1999; Margolin et al., 2020b). This could also result in suboptimal immunogenicity, or the induction of antibodies against epitopes that are not accessible on the wild type virus if glycosylation occludes these regions (Zhou et al., 2017; Margolin et al., 2020b).

It is presently unclear to what extent these different plant-specific glycan structures decorate plant-produced viral glycoproteins, or if under-glycosylation occurs in this context. Therefore, in this study we aimed to define the site-specific glycan occupancy of cleavage-independent heavily glycosylated envelope glycoproteins from HIV and Marburg virus (MARV) that were produced in *Nicotiana benthamiana* plants. These examples were chosen as challenging test cases for the development of plant expression systems capable of producing humanized mimetics of these extensively post-translationally modified viral glycoproteins. A head-to-head comparison of the site-specific glycan occupancy of the proteins was performed with the equivalent mammalian cell-produced proteins. We also determined the glycosylation of plant-produced Epstein-Barr virus (EBV) gp350 and a cleaved HIV Env SOSIP.664 antigen from a previous study (Margolin et al., 2020c), to determine whether the structures observed were common to heavily glycosylated glycoproteins produced in the system.

## Materials and Methods

### Design of Genes for Heterologous Expression in Plants and Mammalian Cells

The design and codon optimization of the synthetic gene sequences for HIV-1 CAP256 SU gp140 NFL, HIV-1 CAP256 SU SOSIP.664, EBV gp350, furin and calreticulin have been previously described (van Diepen et al., 2018; Margolin et al., 2019, 2020c). The MARV glycoprotein antigen (GP) generated in this study was based on the sequence of the Lake Victoria isolate (strain Musoke-80, UniProt accession #P35253). A soluble derivative of the antigen gene was constructed by truncating the ORF after amino acid 648 to remove the transmembrane and cytoplasmic domains. The gene sequence was further modified by replacing the native leader sequence with the heterologous leader peptide heavy chain (LPH) leader peptide (Margolin et al., 2019) or the tissue plasminogen activator (TPA) leader sequence, and replacing the furin cleavage site with a (GGGGS)_2_ linker (van Diepen et al., 2018). A synthetic Kozak sequence (CCACC) was included at the 5’ end of the gene. The MARV glycoprotein coding sequence was synthesized to reflect the preferred human codon usage.

### Construction of Expression Plasmids for Heterologous Glycoprotein Production

Recombinant protein expression was done using the pEAQ-*HT* and pTHpCapR plasmid systems for glycoprotein expression in plants and mammalian cells, respectively (Sainsbury et al., 2009; Tanzer et al., 2011; van Diepen et al., 2018; Margolin et al., 2019). A stably transfected HEK293 cell line was previously generated for production of the cleavage-independent soluble HIV Env gp140 antigen from the CAP256 SU virus (van Diepen et al., 2018). Similarly, recombinant *Agrobacterium tumefaciens* strains encoding the matched HIV Env antigen, the EBV gp350 antigen and the CAP256 SU SOSIP.664 gp140 were previously generated as part of independent studies (Margolin et al., 2019, 2020c). The MARV GPΔTM sequence was assembled in pEAQ-*HT*. The LPH leader was replaced with the TPA leader sequence for expression in mammalian cells and the fusion gene cloned into the pTHpCapR expression plasmid. The recombinant pEAQ-*HT* expression plasmid was electroporated into *A. tumefaciens* AGL1 as described previously (Margolin et al., 2019).

### Recombinant Viral Glycoprotein Production in Plants and Mammalian Cells

All recombinant viral glycoproteins were produced in plants by agroinfiltration of *N. benthamiana* with recombinant *A. tumefaciens* bacteria as described previously (Margolin et al., 2020c). The HIV Env gp140 NFL protein was produced using a stably transfected cell line that was developed as part of an independent study (van Diepen et al., 2019). MARV GPΔTM was expressed by transient transfection of HEK293T cells, under serum-free conditions as previously described (van Diepen et al., 2018). Expression of the MARV GPΔTM protein was verified by western blotting of crude cell lysates using a polyclonal rabbit antibody raised against a synthetic peptide (CDQIKKDEQKEGTGW) in immunized rabbits. The recombinant proteins were purified from either cell culture media or leaf lysate following homogenization. The HIV and MARV glycoproteins were purified by sequential *Galanthus nivalis* lectin (GNL) affinity chromatography and gel filtration, as previously described, whereas the EBV gp350ΔTM protein was purified directly by GNL-affinity chromatography (Margolin et al., 2019; van Diepen et al., 2019). Size exclusion chromatography profiles were normalized by dividing each datapoint by the peak signal and then represented as a proportion of 1. The data was presented as overlayed elution profiles using GraphPad Prism 5 software. The purified proteins were quantified using the Bio-Rad protein *DC* assay. The recombinant antigens were also resolved on 4–12% NativePAGE Bis-Tris gels and stained with Bio-Safe^TM^ Coomassie.

### Site-Specific N-Glycan Analysis of Recombinant Viral Glycoproteins

In order for each glycoprotein to be analyzed by liquid-chromatography-mass spectrometry (LC-MS), three aliquots of purified protein were denatured for 1 h in 50 mM Tris/HCl [pH 8.0], containing 6 M of urea and 5 mM of dithiothreitol (DTT). Next, the proteins were reduced and alkylated by adding 20 mM iodoacetic acid (IAA) and incubating the samples for 1 h in the dark. The samples were then incubated with DTT to remove residual IAA. The alkylated glycoproteins were buffer exchanged into 50 mM Tris/HCl [pH 8.0] and digested separately using trypsin, chymotrypsin (Mass Spectrometry Grade, Promega) and alpha-lytic protease (Sigma-Aldrich) at a ratio of 1:30 (w/w). Following overnight digestion, the resulting peptide/glycopeptides were dried and extracted using C18 Zip-tip (Merck Millipore). After elution, the peptide/glycopeptides were dried again, resuspended in 0.1% formic acid and then analyzed by nanoLC-ESI MS with an Easy-nLC 1200 (Thermo Fisher Scientific) system coupled to a Fusion mass spectrometer (Thermo Fisher Scientific) using higher energy collision-induced dissociation (HCD) fragmentation. Peptides were separated using an EasySpray PepMap RSLC C18 column (75 μm × 75 cm). A trapping column (PepMap 100 C18, 3 μm particle size, 75 μm × 2 cm) was used in line with the LC prior to separation with the analytical column. The LC conditions were as follows: 275 min linear gradient consisting of 0-32% acetonitrile in 0.1% formic acid over 240 min, followed by 35 min of 80% acetonitrile in 0.1% formic acid. The flow rate was set to 300 nl/min. The spray voltage was set to 2.7 kV and the temperature of the heated capillary was set to 40°C. The ion transfer tube temperature was set to 275°C. The scan range was 400-1600 m/z. The HCD collision energy was set to 50%, appropriate for fragmentation of glycopeptide ions. Precursor and fragment detection were performed using an Orbitrap at a resolution MS1 = 100,000. MS2 = 30,000. The automatic gain control (AGC) target for MS1 = 4e5 and MS2 = 5e4 and injection time: MS1 = 50 ms MS2 = 54 ms.

Glycopeptide fragmentation data were extracted from the raw file using Byonic^TM^ and Byologic^TM^ software (Version 3.5; Protein Metrics Inc.). The glycopeptide fragmentation data were evaluated manually for each glycopeptide; the peptide was scored as true-positive when the correct b and y fragment ions were observed along with oxonium ions corresponding to the glycan identified. The MS data was searched using the Protein Metrics’ N-glycan libraries: for mammalian expression the 309 N-glycan library was used and for material produced in plants the 52 plant library was used. The relative amounts of glycan at each site, as well as the unoccupied proportion, were determined by comparing the extracted chromatographic areas for different glycotypes with an identical peptide sequence. All charge states for a single glycopeptide were summed. The precursor mass tolerance was set at 4 ppm and 10 ppm for fragments. A 1% false discovery rate (FDR) was applied. The relative amounts of each glycoform at each site, as well as the unoccupied proportion, were determined by comparing the extracted ion chromatographic areas for different glycopeptides with an identical peptide sequence. Glycans are categorized according to the detected compositions by LC-MS. Compositions containing Hex(10-12) HexNAc(2) were categorized as M9Glc1-3 and Hex(9-5) HexNAc(2) as M9-M5. These are classified as oligomannose-type glycans and are colored green. Hybrid-type glycans contain compositions consisting of Hex(5)HexNAc(3)X or Hex(6)HexNAc(4)X. Remaining glycan compositions were assigned as complex-type. Any composition Hex(3) HexNAc(2) or smaller is classified as truncated. The proportion of unoccupied N-linked glycan sites at each site are colored gray.

## Results

### Viral Glycoprotein Selection and Antigen Design

**FIGURE 1:**
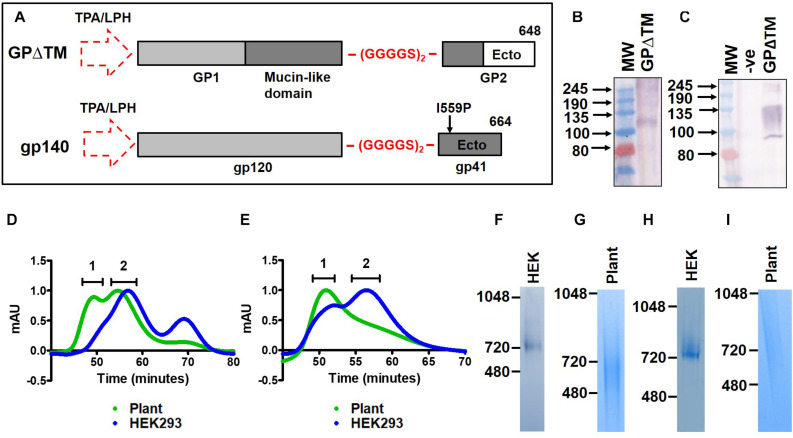
Design and purification of recombinant viral glycoproteins in plants. **(A)** Schematic of the synthetic MARV GPΔTM (top) and HIV Env gp140 (bottom) antigen sequences. The natural signal peptide was replaced with the LPH or TPA leader peptides (dotted arrow) for expression in plants and mammalian cells respectively. A flexible linker peptide, (GGGGS)_2_, was inserted in place of the furin cleavage site and the transmembrane and cytoplasmic domains of the proteins were removed. The location of the GP_1_ and GP_2_ subunits, and the mucin-like domain are indicated for MARV GPΔTM. The gp120 and gp41 subunits are similarly shown for HIV Env gp140. A stabilizing isoleucine to proline mutation was also included in the HIV Env gp140 antigen (I559P). The amino acids where the proteins were truncated are indicated as 648 and 664 for MARV and HIV, respectively. **(B)** Western blotting of plant homogenate to detect expression of recombinant MARV GPΔTM. **(C)** Western blotting to detect expression of MARV GPΔTM in transfected mammalian cell lysate. In both panels **(B,C)**, the recombinant protein was detected using a polyclonal rabbit antibody raised against a synthetic peptide from the glycoprotein. **(D,E)** Overlayed S200 elution profiles of recombinant plant-produced and mammalian cell-derived HIV Env gp140 NFL and MARV GPΔTM, respectively. The plant-produced protein is shown in green whereas the mammalian cell-derived protein is indicated in blue. 1 = aggregate peak, 2 = putative trimer peak. Coomassie-stained BN-PAGE gels for the pooled trimer fractions are shown in panels **(F–I)** for mammalian cell-produced HIV Env gp140 (HEK), plant-plant produced gp140 (plant), mammalian cell-derived MARV GPΔTM and plant-produced MARV GPΔTM, respectively. (-ve = untransfected cell lysate, Ecto = Ectodomain, TM = transmembrane region, CT = cytoplasmic tail).

Viral envelope glycoproteins from HIV-1, EBV, and MARV were selected for this study as they are all extensively glycosylated and are therefore ideal models to interrogate the impact of the plant cellular machinery on glycosylation. Both HIV gp140 and EBV gp350ΔTM were successfully expressed in plants as part of a previous study exploring the impact of co-expressing human chaperones on viral glycoprotein production in *N. benthamiana* (Margolin et al., 2020c). MARV is a re-emerging virus with a high probability of causing disease outbreaks in humans, prompting the suitability of plant-based expression to be explored for the development of a vaccine against a prototype emerging filovirus (Letko et al., 2020). MARV and HIV glycoproteins were both expressed as soluble cleavage-independent antigens for ease of production and recovery (Figure 1A) using previously described approaches (Margolin et al., 2019, 2020c; van Diepen et al., 2019).

### Transient Expression of MARV GPΔTM in Plants and Mammalian Cells

Marburg virus GPΔTM was transiently expressed in plants and mammalian cells by agroinfiltration and transfection, respectively. Western blotting of crude extract from agroinfiltrated leaves yielded a product of ∼115-125 kDa and some diffuse higher molecular weight products which were not well resolved by SDS-PAGE (Figure 1B). Western blotting of lysate from transfected HEK293 cells produced a similar product, although the protein appeared slightly larger and the signal was fairly diffuse (Figure 1C). An additional product of <100 kDa was observed for the mammalian cell-produced protein which may have arisen from intracellular processing. Expression of the recombinant HIV Env gp140 antigens and EBV g350ΔTM were both previously described (Margolin et al., 2019, 2020c; van Diepen et al., 2019).

### Purification of Soluble MARV and HIV Glycoproteins

The soluble glycoproteins (HIV Env gp140 and MARV GPΔTM) were captured by GNL affinity chromatography and then fractionated by gel filtration, to recover trimeric glycoproteins and to exclude non-trimeric protein species (Figures 1D,E). The size exclusion chromatography elution profiles for the plant-produced and mammalian cell-derived glycoproteins were overlayed to compare the relative abundance of the different protein species in both systems. Both glycoproteins exhibited a marked increase in higher molecular weight aggregates when produced in plants as reflected in the profile shifting toward the left (Figures 1D,E). While the plant-produced HIV Env gp140 exhibited a peak of the expected size for trimers (Figure 1D), the plant-produced MARV GPΔTM did not yield a defined peak as expected (Figure 1E). Instead, a diffuse shoulder was observed suggesting that the purified protein was highly heterogenous, even after purification.

Coomassie-stained BN-PAGE gels mirrored the observations from size exclusion chromatography. The pooled trimer peak derived from mammalian cells yielded a defined band of ∼720 kDa for both HIV Env gp140 (Figure 1F) and MARV GPΔTM (Figure 1H), which is consistent with our previous observations for HIV (van Diepen et al., 2019) and published accounts for filovirus glycoprotein trimers (Rutten et al., 2020). In contrast, Coomassie staining of the pooled fractions corresponding to the plant-derived glycoprotein trimers yielded a diffuse signal (Figures 1G,I).

### Aberrant Glycosylation of HIV Env Using *N. benthamiana* as a Production System

**FIGURE 2:**
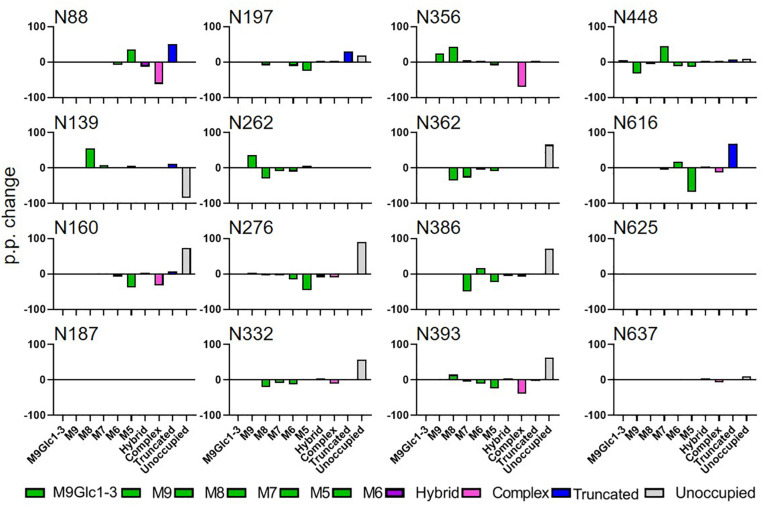
Comparison of site-specific glycosylation of HIV Env gp140 NFL when produce in plants or mammalian cells. The data represent the percentage point change in glycosylation when the protein was expressed in *N. benthamiana* compared to HEK293, so a positive value represents a category of glycan that is enriched on material produced in *N. benthamiana*. Glycans are categorized according to the detected compositions by LC-MS. Compositions containing Hex(10-12) HexNAc(2) were categorized as M9Glc1-3 and Hex(9-5) HexNAc(2) as M9-M5. These are classified as oligomannose-type glycans and are colored green. Hybrid-type glycans contain compositions consisting of Hex(5)HexNAc(3)X or Hex(6)HexNAc(4)X. Remaining glycan compositions were assigned as complex-type. Any composition Hex(3) HexNAc(2) or smaller is classified as truncated. The proportion of potential N-linked glycans which are classified as unocupied are coloured grey.

To determine the site-specific changes in glycosylation of HIV-1 Env when using *N. benthamiana* as the expression system we applied LC-MS to the purified proteins (Figure 2). The data is displayed as the percentage point change in site-specific glycosylation when the protein is expressed using *N. benthamiana*, so a positive value represents a category which is more abundant in *N. benthamiana* compared to mammalian produced protein. This methodology has been used previously to compare and contrast the glycosylation of the viral glycoprotein from BG505 and other HIV strains (Behrens et al., 2016; Struwe et al., 2018). There are distinct glycan processing states on HIV Env which form key epitopes for broadly neutralizing antibodies. The early glycan processing pathway in both mammals and plants is conserved and involves the attachment and subsequent trimming of oligomannose-type glycans in the ER and early Golgi apparatus (Montero-Morales and Steinkellner, 2018). The density of N-linked glycans on HIV Env perturb this process, and the resultant mature protein contains remnants of these under-processed glycans. These oligomannose-type glycans can form epitopes for broadly neutralizing antibodies that target several regions of the Env spike. Two key regions for antibody recognition are the intrinsic mannose patch (IMP), focused around the N332 supersite of vulnerability, and the trimer-associated patch (TAMP), with the N160 glycans of the V1/V2 region forming a key part of several antibody epitopes (Behrens and Crispin, 2017). The TAMP is only present when correctly processed and cleaved Env is analyzed, and forms at the trimer interface and the apex of the protein (Behrens et al., 2017). These two key regions of oligomannose-type glycans are key for immunogen design efforts (Haynes et al., 2019).

When comparing the glycosylation of CAP256 SU gp140 expressed in mammalian and plant cells the distribution and processing of these oligomannose-type glycans is affected. Sites that form the IMP such as N332 and N386 show a decrease in oligomannose-type glycans (40 and 55 percentage points, respectively) when *N. benthamiana* is used as the expression system (Supplementary Table 1). The TAMP is also disrupted with a similar reduction in oligomannose-type glycans at N160 and N197. Oligomannose-type glycans increase in abundance at N356, however this site appears more fully processed in HEK293 cells.

The reason for the decrease in oligomannose-type glycans at key bnAb recognition sites is the near universal increase in potential N-linked glycosylation sites that lack post-translational modifications. Sites N160, N197, N276, N332, N362, N386, N393, N447, and N637 all show an increase in the proportion of unoccupied glycan sites. This represents an asparagine of an NxS/T sequon which has the capacity for the addition of an N-linked glycan, but where the glycosylation machinery has not attached a glycan. Recombinant expression systems are suboptimal for recapitulating the high occupancy of viral-derived Env, especially on gp41, and this has also been observed in mammalian expression systems, albeit to a lesser extent (Derking et al., 2021).

This can be seen on CAP256 SU gp140 expressed in both HEK293 and *N. benthamiana*, for example at N625 (Supplementary Table 1). The presence of these so-called glycan holes has been shown to induce non-neutralizing antibody responses during immunizations which do not protect from HIV acquisition (Crooks et al., 2015; Zhou et al., 2017). The CAP256 SU gp140 expressed using *N. benthamiana* contains the same glycan holes as with HEK293 cells, however, the presence of additional holes at sites such as N332 will likely generate detrimental antibody responses when used in immunizations. Finally, at three sites on CAP256 SU gp140 from *N. benthamiana*, N88, N197 and N616, a substantial increase in smaller glycan structures were observed (ranging from a single hexosamine to Man_3_GlcNAc_2_) which were not observed on mammalian proteins.

### Incomplete Processing of Complex Glycans on MARV GPΔTM Expressed in *N. benthamiana* Compared to Mammalian Cells

**FIGURE 3:**
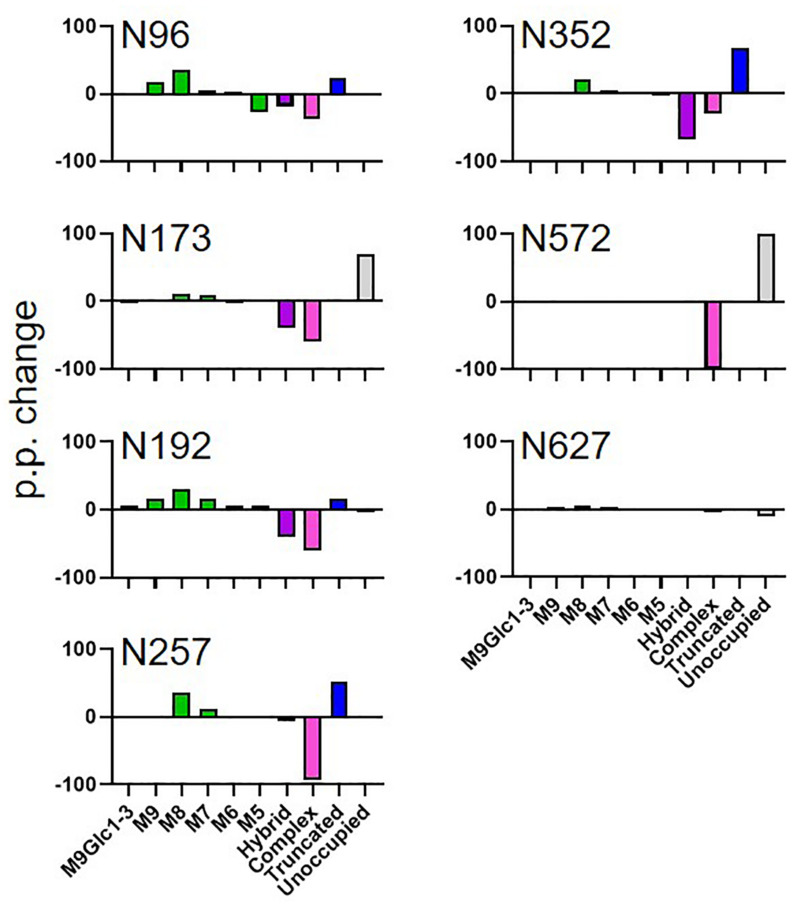
Comparison of MARV GPΔTM glycosylation when the protein was expressed in plants and mammalian cells. The data represent the percentage point change in glycosylation when the protein was expressed in *N. benthamiana* compared to HEK293, so a positive value represents a category of glycan that is enriched on material produced in *N. benthamiana*. Glycans are categorized according to the detected compositions by LC-MS. Compositions containing Hex(10-12) HexNAc(2) were categorized as M9Glc1-3 and Hex(9-5) HexNAc(2) as M9-M5. These are classified as oligomannose-type glycans and are colored green. Hybrid-type glycans contain compositions consisting of Hex(5)HexNAc(3)X or Hex(6)HexNAc(4)X. Remaining glycan compositions were assigned as complex-type. Any composition Hex(3) HexNAc(2) or smaller is classified as truncated. The proportion of potential N-linked glycans which are classified as unocupied are coloured grey.

To investigate whether the observed changes in glycosylation were specific to HIV Env, we performed a comparative analysis of MARV GPΔTM produced in HEK293 and *N benthamiana*. Data of sufficient quality could only be obtained for 8 sites with the full list of sites shown in Supplementary Table 2. As with HIV Env, the site-specific glycosylation of MARV GPΔTM varied extensively between HEK293 and *N. benthamiana* (Figure 3). For the HEK293 cell-produced protein, several sites displayed extensive glycan processing typical of mammalian glycoproteins. Interestingly, a high proportions of hybrid-type glycans were observed, which are named as such because they contain one arm which is fully processed and one arm presenting terminal mannose residues. Importantly, both the hybrid and complex-type glycans observed on HEK293 MARV GP were not present on material produced in *N. benthamiana*. This includes mature glycans modified with xylose which were included in the library used to search the LC-MS data. Instead, there was a global increase of oligomannose-type glycans, for example at N96 in mammalian cells where 41% of the glycans were oligomannose-type. In contrast this increased to 71% when produced in plants, at 30 percentage point increase (Figure 3 and Supplementary Table 2). The presence of elevated amounts of pauciglycan structures was also observed at five sites, with pauciglycans consisting of 20% of the averaged compositions of all sites obtained by LC-MS. Sites N173 and N572 also lacked glycans on 70 and 100% of the sites, whereas for HEK293 these sites were fully occupied. The higher levels of oligomannose-type glycans observed for MARV GPΔTM is likely due to the cell line used for protein production. In the case of HIV Env, the presence of oligomannose-type glycans is due to steric clashes inhibiting the ability of glycan processing enzymes whereas the lack of oligomannose-type glycans on MARV GPΔTM suggests a differential mechanism. The glycan processing machinery in plants may be unable to process such extensive glycosylation to the same extent as HEK293 cells.

In order to determine if this glycosylation signature was specific to the proteins in question, or rather a feature of producing heavily glycosylated glycoproteins in plants, the site-specific glycosylation of previously produced EBV gp350 and CAP256 SU gp140 SOSIP.664 (Margolin et al., 2020c) were also determined (Table 1). Both antigens displayed a similar glycosylation profile comprising of large amounts of oligomannose-type glycans, low levels of complex glycans and considerable amounts of paucimannosidic glycans. Similar to the plant-produced MARV GPΔTM and HIV Env gp140 antigens, a large amount of under-glycosylation was observed. Interestingly for EBV gp350 and CAP256 SU gp140 SOSIP produced in plants, sites displayed glucosylated Man_9_GlcNAc_2_ (categorized as M9Glc1-3 in Table 1). This precursor oligosaccharide is present during the early ER folding stages and the removal of the glucose monosaccharides acts as a checkpoint to signal that the protein is correctly folded. The presence of these glycans in the resultant purified protein is unusual, given that 0% of the glycans from both CAP256 SU gp140 and MARV GPΔTM produced in HEK293 cells contained these moeities. These observations are consistent with the increase in oligomannose-type glycans observed on MARV GPΔTM suggesting that immaturely processed glycoproteins are being released from the ER during homogenization of plant material and purified.

**TABLE 1:**
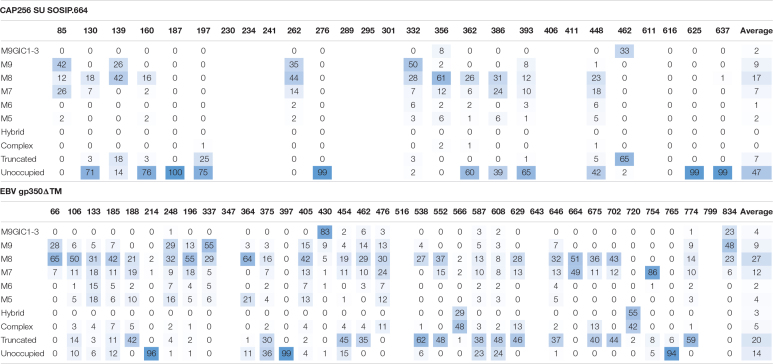
Site-specific glycosylation of plant produced CAP256 SU gp140 SOSIP.664 and EBV gp350ΔTM.

*Different tints of blue, from dark through to light, indicate the highest through to the lowest numbers in the table.*

## Discussion

Viral glycoprotein-based subunit vaccines are amongst the most complex recombinant proteins that are being pursued in plant expression systems (Margolin et al., 2018). These proteins have intricate architectures with extensive post-translational modifications, and are heavily reliant on the host cellular machinery to support their maturation. However, some of these maturation events may not occur optimally in plants, necessitating molecular engineering of the host machinery to support the synthesis of these proteins (Margolin et al., 2020b,e). Approaches that have been successfully implemented to increase glycoprotein yields are co-expression of human chaperones, and ectopic expression of the protease furin to support proteolytic processing in plants (Margolin et al., 2020c). Considerable success in this regard has been reported for the production of antibodies and other less complex glycoproteins (Montero-Morales and Steinkellner, 2018), but it is apparent that continued development of plant-based expression systems will be required for the production of complex viral glycoproteins that recapitulate the post-translational processing of mammalian production systems.

Host-derived glycosylation is central to glycoprotein folding and regulates the interaction of the proteins with ER-resident chaperones (Watanabe et al., 2019). In addition, these glycans also determine the immunogenicity of the glycoprotein in natural infection or following vaccination (Dalziel et al., 2014; Watanabe et al., 2019). Therefore, it may be necessary to reproduce the native glycosylation of many viral glycoproteins to elicit appropriate immune responses in the context of vaccination. As a starting point to develop approaches to produce natively glycosylated viral glycoproteins in plants, we interrogated the site-specific glycosylation of recombinant plant-produced MARV and HIV Env glycoproteins and compared them to the equivalent proteins produced in mammalian cells. We also determined the glycosylation of EBV gp350ΔTM and HIV Env gp140 SOSIP.664 produced in plants in a previous study (Margolin et al., 2020c).

Building on previous work to express HIV Env gp140 in plants and mammalian cells (van Diepen et al., 2018, 2019; Margolin et al., 2019, 2020c), we produced a soluble MARV glycoprotein in both systems. This is to our knowledge the first report describing the expression of this filoviral glycoprotein in a plant expression system, and adds to an increasing number of viral glycoproteins that have been expressed in plants. The HIV and MARV glycoproteins were purified by sequential affinity chromatography and gel filtration steps, and the size exclusion elution profiles were overlayed with the equivalent mammalian cell-produced proteins for comparison. Whilst the HIV Env gp140 antigen produced a peak that is consistent with the expected size for trimeric Env, the MARV GPΔTM protein yielded a diffuse shoulder, suggesting high levels of heterogeneity for the recombinant protein. In both cases, there was a striking shift of the plant-produced protein toward the left of the profile indicating an increase in size. This corresponds to increased aggregation in the system, which is consistent with previous reports describing unresolved higher molecular weight products following the expression of HIV Env gp140 antigens in plants (Rosenberg et al., 2013; Margolin et al., 2019).

Given the extensive under-glycosylation of the protein observed in this study it is likely that these aggregates comprise of aberrantly folded protein species. This is also supported by the presence of glucosylated mannose species that were observed. Whilst several studies have reported under-glycosylation in a range of plant-produced proteins (Jarczowski et al., 2016; Castilho et al., 2018; Singh et al., 2020), this is the most extensive under-glycosylation that has been reported to date. This observation may account for the inferior immunogenicity of the plant-produced HIV gp140 antigen compared to the mammalian cell-produced protein that was observed in a previous study, where the trimer elicited high titers of binding antibodies in immunized rabbits but did not induce any appreciable autologous neutralizing antibodies (Margolin et al., 2019). If these antibodies were raised against epitopes that were shielded by glycans on the wild type virus, it would explain why they failed to neutralize. It is well established that antibodies targeting holes in the glycan shield are readily induced against the HIV Env glycoprotein, and therefore reproducing the glycan shield on trimer immunogens is critical to prevent undesired off-target immune responses (Zhou et al., 2017; Crispin et al., 2018; Ringe et al., 2019). Glycans can also constitute important epitopes targeted by neutralizing antibodies, and in the absence of glycosylation these epitopes would not be reproduced (Crispin et al., 2018). The N160 glycan at the apex of the trimer, for example, is almost completely unoccupied in the plant-produced antigen, and therefore the protein is not expected to exhibit appreciable binding by PG-9 and other related antibodies that recognize this epitope (McLellan et al., 2011). Under-glycosylation was similarly observed at the N332 glycan site which is commonly targeted by broadly neutralizing antibodies during natural infection (Sok et al., 2016). Future work should compare the reactivity of plant-produced and mammalian cell-derived proteins with prototype monoclonal antibodies from natural infection. These antibodies could serve as useful tools to further investigate how closely these plant-produced antigens resemble their mammalian cell-produced counterparts, and will help establish if host engineering approaches improve their folding and antigenicity.

Based on the work described here, and in other recent studies of plant-produced enzymes and antibodies, it seems likely that under-glycosylation of heterologous proteins may be more common than was previously appreciated (Castilho et al., 2018; Göritzer et al., 2020). Given the central role of glycosylation in protein folding, this could be an important contributory factor to inefficient production of certain viral glycoproteins in the system, and could be expected to pose a similar challenge for other similarly glycosylated target proteins (Margolin et al., 2018). The molecular basis for under-glycosylation in the system is not well understood but it has been suggested that this is related to the distinct recognition preferences of the plant oligosaccaryltransferase complex (Margolin et al., 2020e). It is also probably a consequence of the purification process where homogenization of plant tissue will liberate proteins from all stages of the secretory pathway, including those that have not yet been properly glycosylated. Encouragingly, it has recently been shown that the co-expression of *Leishmania major* LmSTT3D increased the glycan occupancy of several model proteins in *N. benthamiana* (Castilho et al., 2018; Göritzer et al., 2020).

The almost complete absence of plant-specific complex glycans in the purified proteins is an unexpected observation, and contrasts with other reports describing plant-produced viral glycoproteins from influenza virus and HIV-1 (Rosenberg et al., 2013; Le Mauff et al., 2015). However, it is difficult to compare these to the current study due to differences in glycoprotein complexity and different antigen design strategies: both glycoproteins produced in this study contain considerably more N-glycan sequons than influenza HA which only contains six N-glycan sites, and therefore the burden on the host cellular machinery is expected to be commensurately greater (Le Mauff et al., 2015). The HIV-1 Env trimer described in this study was also engineered to preserve the structure, whereas the previously described glycoprotein was modified to remove the cleavage site, fusion peptide and immunodominant region of gp41 (Rosenberg et al., 2013). These alterations are expected to have impacted protein folding, and based on subsequent insights into the protein structure, would be likely to have compromised the folding of the glycoprotein (Ringe et al., 2013). It is also plausible that the processing by host mannosidases could be less efficient in plants resulting in poor formation of complex glycans. These enzymes may be less abundant in plants than mammalian cells as the requirement for glycosylation is often considerably less.

Although the levels of truncated and paucimannose-type glycans following expression in plants is surprising, it is not unprecedented. Similar observations have been reported for other heterologous plant-produced proteins, such as α1-antitrypsin (Castilho et al., 2014), IgA (Dicker et al., 2016), and bovine follicle stimulation hormone (Dirnberger et al., 2001). These truncated glycans arise from the removal of *N*-acetylglucosamines from the non-reducing end by β-hexosaminidases (HEXOs) (Liebminger et al., 2011). The responsible enzymes are localized to vacuoles and the plasma membrane in *N. benthamiana*, and previous work has shown that their downregulation by RNA interference increased the levels of intact complex-type glycans (Shin et al., 2017). These structures do not naturally occur on viral glycoproteins and therefore this approach will be necessary to eliminate this undesirable processing *in planta*.

In conclusion, we have delineated the site-specific glycosylation of several model human viral glycoproteins that were produced in *N. benthamiana*, and identified key constraints in the host glycosylation machinery. This work adds to a growing body of evidence suggesting that remodeling the secretory pathway may be necessary to support the production of complex pharmaceutical targets in the plant expression system, and provides a rational starting point to produce recombinant glycoproteins with humanized glycosylation in plants (Margolin et al., 2020e). The data presented here has important implications for plant molecular farming of viral glycoproteins and suggests that under-glycosylation may be more widespread than previously realized.

## Data Availability Statement

The original contributions presented in the study are included in the article/Supplementary Material, further inquiries can be directed to the corresponding author/s.

## Author Contributions

EM, JA, and MC conceptualized the study. EM and RC designed the gene sequences and conceived the cloning strategy. EM and MV conducted protein expression in plants. EM, MD, and PX conducted protein expression in mammalian cells. JA carried out the site-specific glycosylation. A-LW and RC supervised mammalian cell culture aspects of the project. ER and AM supervised plant-based protein expression component of the project. EM and JA drafted the manuscript. Funding for the project in Cape Town was obtained by EM, A-LW and ER. All authors contributed to data analysis and reviewed the manuscript.

## Conflict of Interest

EM, RC, AM, AL-W, and ER have filed patent applications describing the development of approaches to support production of glycoproteins in plants including US 2019/0337994 A1, WO 2018 220595 A1, PA174002_PCT, and PA2106659.4. The remaining author declares that the research was conducted in the absence of any commercial or financial relationships that could be construed as a potential conflict of interest.

## Supplementary Material

Supplementary Table 1 Comparison of site-specific glycosylation of HIV Env gp140 NFL when produce in plants or mammalian cells. The glycosylation profile of the recombinant protein is shown when the protein was expressed in *N. benthamiana* **(A)** and HEK293 cells **(B)**. The change in glycosylation that occurs when the protein is produced in plants is shown in **(C)**.

Supplementary Table 2 Comparison of MARV GPΔTM glycosylation when the protein was expressed in plants and mammalian cells. The glycosylation of the glycoprotein is shown when the protein was expressed in *N. benthamiana* **(A)** and in HEK293 cells **(B)**. The change in glycosylation that occurs when the protein was produced in *N. benthamiana* is shown in **(C)**. (m5-m12 = high-mannose, pauci = paucimannosidic). In each cases the numbers on *X* axis of the Figure indicate the position of a putative N-glycan sequon. (m5-m12 = high-mannose, pauci = paucimannosidic). In each case the number above the tables indicates the position of a putative N-glycan sequon. Numbers in the table indicate the relative proportion of each glycoforms at this site. The table to the right summarizes the relative abundance of each glycan species considering all the individual sites together.
