# Supplementary material for: Site-Specific Glycosylation of Recombinant Viral Glycoproteins Produced in Nicotiana benthamiana
Source: Front Plant Sci. 2021 Jul 22;12:709344. doi: 10.3389/fpls.2021.709344 (PMC8341435; doi:10.3389/fpls.2021.709344)
Supplement: Supplementary Table 1 — Comparison of site-specific glycosylation of HIV Env gp140 NFL when produce in plants or mammalian cells. The glycosylation profile of the recombinant protein is shown when the protein was expressed in N. benthamiana (A) and HEK293 cells (B). The change in glycosylation that occurs when the protein is produced in plants is shown in (C). [file Presentation_1.PPTX]

## Slide 1
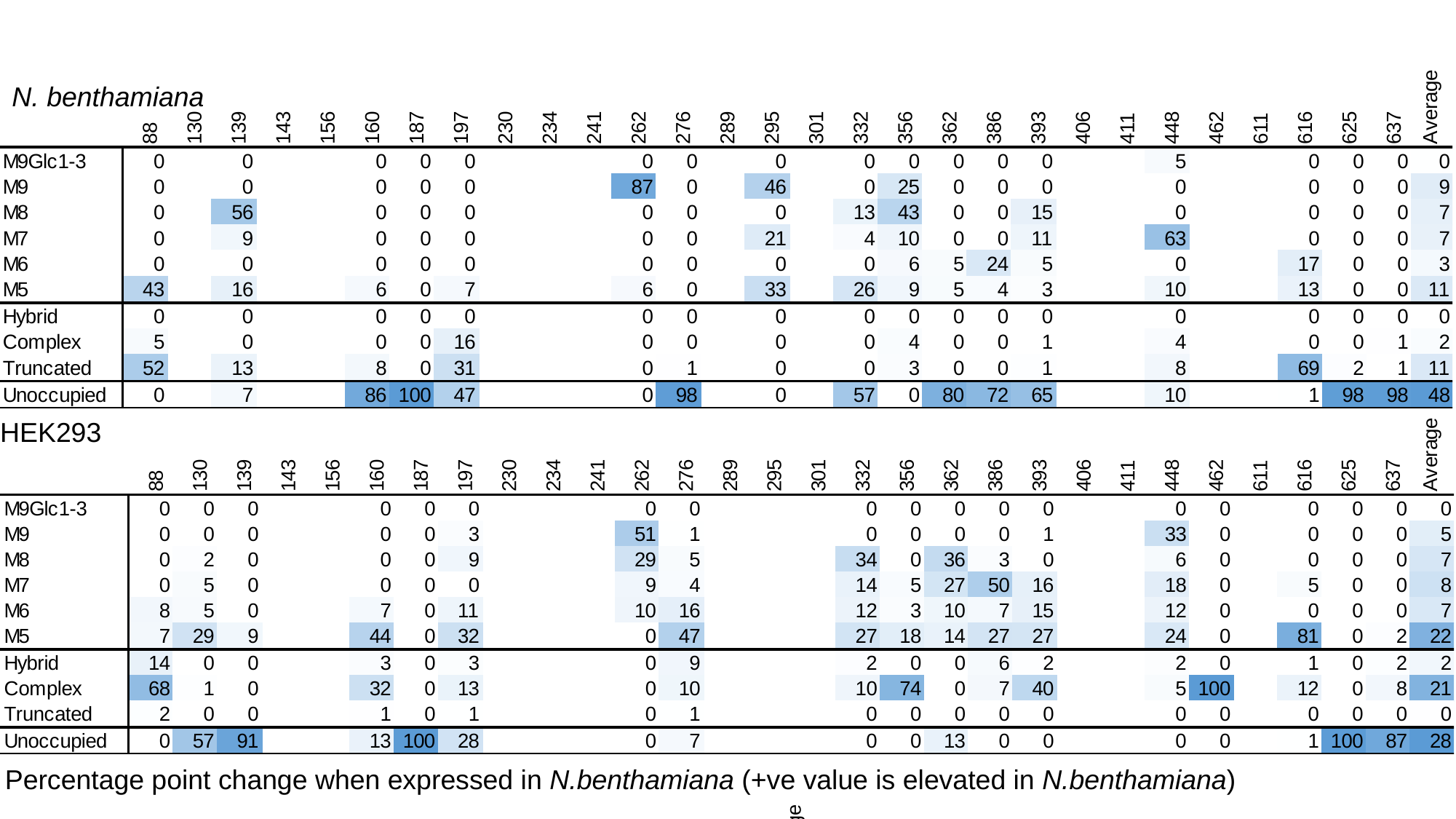

Percentage point change when expressed in N.benthamiana (+ve value is elevated in N.benthamiana)
N. benthamiana
HEK293
