# Supplementary material for: Site-Specific Glycosylation of Recombinant Viral Glycoproteins Produced in Nicotiana benthamiana
Source: Front Plant Sci. 2021 Jul 22;12:709344. doi: 10.3389/fpls.2021.709344 (PMC8341435; doi:10.3389/fpls.2021.709344)
Supplement: Supplementary Table 2 — Comparison of MARV GPΔTM glycosylation when the protein was expressed in plants and mammalian cells. The glycosylation of the glycoprotein is shown when the protein was expressed in N. benthamiana (A) and in HEK293 cells (B). The change in glycosylation that occurs when the protein was produced in N. benthamiana is shown in (C). (m5-m12 = high-mannose, pauci = paucimannosidic). In each cases the numbers on X axis of the Figure indicate the position of a putative N-glycan sequon. (m5-m12 = high-mannose, pauci = paucimannosidic). In each case the number above the tables indicates the position of a putative N-glycan sequon. Numbers in the table indicate the relative proportion of each glycoforms at this site. The table to the right summarizes the relative abundance of each glycan species considering all the individual sites together. [file Presentation_2.PPTX]

## Slide 1
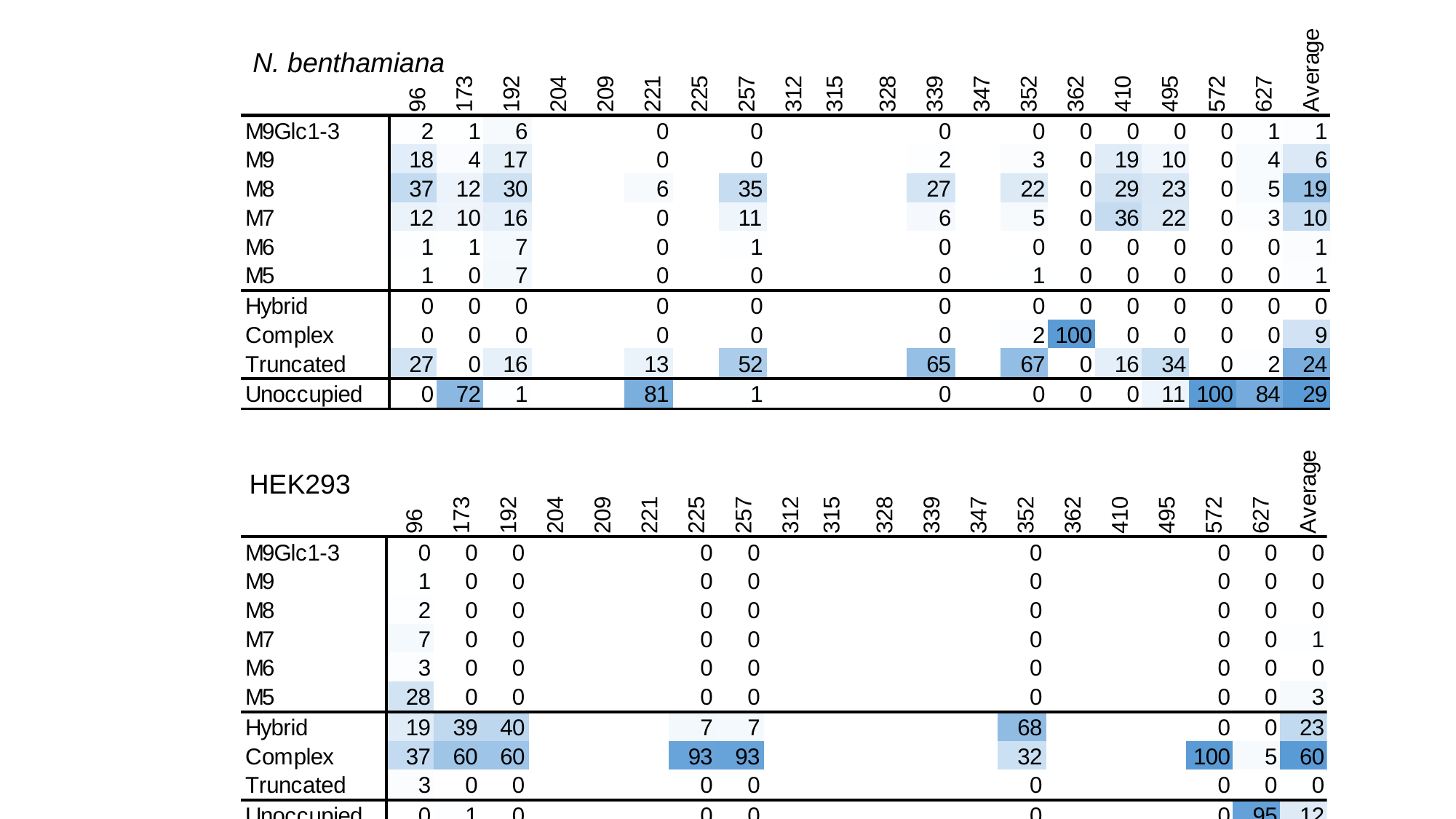

N. benthamiana
HEK293
Percentage point change when expressed in N.benthamiana (+ve value is elevated in N.benthamiana)
